# Genome analysis of peeling archival cytology samples detects driver mutations in lung cancer

**DOI:** 10.1002/cam4.3089

**Published:** 2020-04-29

**Authors:** Kei Kunimasa, Yosuke Hirotsu, Kenji Amemiya, Yuki Nagakubo, Taichiro Goto, Yoshihiro Miyashita, Yumiko Kakizaki, Toshiharu Tsutsui, Sotaro Otake, Hiroaki Kobayashi, Rumi Higuchi, Kie Inomata, Takashi Kumagai, Hitoshi Mochizuki, Harumi Nakamura, Shin‐ichi Nakatsuka, Kazumi Nishino, Fumio Imamura, Toru Kumagai, Toshio Oyama, Masao Omata

**Affiliations:** ^1^ Department of Thoracic Oncology Osaka International Cancer Institute Osaka Japan; ^2^ Genome Analysis Center Yamanashi Central Hospital Yamanashi Japan; ^3^ Department of Surgery School of Medicine Keio University Tokyo Japan; ^4^ Lung Cancer and Respiratory Disease Center Yamanashi Central Hospital Yamanashi Japan; ^5^ Department of Diagnostic Pathology and Cytology Osaka International Cancer Institute Osaka Japan; ^6^ Department of Pathology Yamanashi Central Hospital Yamanashi Japan; ^7^ The University of Tokyo Tokyo Japan

**Keywords:** cytology, driver mutation, fusion gene, lung cancer, next‐generation sequence

## Abstract

**Introductions:**

When tumor tissue samples are unavailable to search for actionable driver mutations, archival cytology samples can be useful. We investigate whether archival cytology samples can yield reliable genomic information compared to corresponding formalin‐fixed paraffin‐embedded (FFPE) tumor samples.

**Patients and Methods:**

Pretreatment class V archival cytology samples with adequate tumor cells were selected from 172 lung cancer patients. The genomic profiles of the primary lung tumors have been analyzed through whole‐exome regions of 53 genes. We compared the genomic profiles based on the oncogenicity and variant allele frequency (VAF) between the archival cytology and the corresponding primary tumors. We also analyzed the genomic profiles of serial cytological samples during the treatment of EGFR‐TKI.

**Results:**

A total of 43 patients were analyzed with the paired samples for DNA mutations and other three patients were analyzed for their fusion genes. A total of 672 mutations were detected. Of those, 106 mutations (15.8%) were shared with both samples. Sixty of seventy‐seven (77.9%) shared mutations were oncogenic or likely oncogenic mutations with VAF ≧10%. As high as 90% (9/10) actionable driver mutations and *ALK* and *ROS1* fusion genes were successfully detected from archival cytology samples. Sequential analysis revealed the dynamic changes in EGFR‐TKI‐resistant mutation (*EGFR* p.T790M) during the course of treatment.

**Conclusion:**

Archival cytology sample with adequate tumor cells can yield genetic information compared to the primary tumors. If tumor tissue samples are unavailable, we can use archival cytology samples to search for actionable driver mutations.

## Graphical abstract

Limited quantity of tumor tissue is a matter to obtain full genomic information in lung cancer. Archival cytology samples can yield genetic information of the correspondent primary tumor. 90% targetable driver mutations were successfully detected from archival cytology samples.

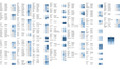

## 1 INTRODUCTION

Lung cancer is the leading cause of cancer‐related deaths world‐wide.^1^ In the past decades, genetic and genomic profiling in non–small‐cell lung cancer (NSCLC) has progressed. The understanding of underlying molecular mechanisms of the disease has improved and developed strategies for targeted therapies to the driver gene mutation.^2^, ^3^ The identification of actionable driver mutations; activating epithelial growth factor receptor (*EGFR*) mutations, anaplastic lymphoma kinase (*ALK*) fusion genes and c‐ROS oncogene 1 (*ROS1*) fusion genes has opened up new treatment for advanced NSCLC patients harboring those mutations.^4^, ^5^ Furthermore, there is a strong recommendation to perform a broader genome profiling for detection of rare mutations; *BRAF, KRAS*, *MET* mutations, and *RET* fusion genes, for which the corresponding targeted therapies are available or suitable for off‐label treatment in clinical trials.^6^, ^7^ At the same time, the identification of those patients who have the actionable driver mutations has made to an ongoing effort to identify the genetic biomarkers as soon as possible in clinical practice. With the advance of next‐generation sequencing (NGS), also known as high‐throughput sequencing, in clinical diagnostics has revolutionized the clinical medicine including the field of lung cancer.^8^ NGS testing enables us to overcome many of the shortcomings of direct sequencings and allele‐specific molecular testing.

To conduct NGS testing with high success rate, we need more clinical sample which contain sufficient amounts of tumor cells. In clinical practice, surgical specimen is one of the ideal samples to obtain high quality and high amount DNA/RNA for the NGS testing. However, especially in cases with advanced lung cancer patients, sampling procedure mainly depends on less invasive bronchoscopy than surgical procedures in clinical setting. It is difficult to obtain sufficient amount of tumor tissue by bronchoscopy to search for actionable driver mutations. Although the feasibility of small samples obtained by bronchoscopy for successful NGS testing has been shown,^9^ the amount of tumor sample is still a large matter of concern. More than one biomarker search is now required, and each time a paraffin block is cut for submitting tumor sample, the block is worn out for revealing the tumor. Surgical biopsy specimens are rarely obtained during the course of treatment especially in treatment for advanced lung cancer patients. Increasing number of clinical molecular tests put a challenge to the limited amount of tumor samples obtained from less invasive clinical procedures and versatility of cytology samples provides multiple options for performing increasing molecular tests.^10^, ^11^, ^12^

The cytology samples for NGS testing showed comparable sequencing performance compared with samples obtained by surgical procedure.^13^ In the study, fine needle aspiration (FNA)‐obtained samples were selected for NGS testing and various types of cancer patients were enrolled. In clinical practice of lung cancer diagnostics, transbronchial biopsy with or without endobronchial ultrasound is a major procedure to diagnose pulmonary lesions and cytology samples are obtained not only from FNA but also from cytology brushes and biopsy forceps.^14^, ^15^ Here, we aim to evaluate the feasibility of applying NGS testing to the genetic analysis of archival cytology samples routinely obtained from clinical practice including bronchoscopy in a clinical molecular diagnostic laboratory.

## 2 MATERIALS AND METHODS

### 2.1 Case and sample selection

In this study, we selected cytology samples routinely obtained from bronchoscopy or surgery for 172 consecutive lung cancer patients with known mutation status for 53 lung cancer relevant genes between January 2014 and January 2018. All bronchoscopy investigations were performed for definite diagnosis to investigate abnormal shadows in lung fields. Any treatment was not introduced before bronchoscopy. All cytology samples were made from disposable bronchial cytology brushes, bronchial wash after transbronchial biopsy, FNA or preresection pleural lavage. Cytology smears were stained with Papanicolaou (ethanol‐fixed) or May‐Giemsa (air‐dried methanol‐fixed). All slides were reviewed by a pathologists (TO) and a cytologist (KA) to mark the tumor‐rich areas and the amounts of tumor cells. We selected samples harboring the total number of tumor cells was estimated at least 1000 cells in the slide.^16^, ^17^

### 2.2 Extraction method; peeling off tumor cells from slides

For cytology slides containing sufficient tumor cells more than 1000 tumor cells with an assembled mass, the slide was immersed in xylene to remove the cover glass. Direct scraping using a razor blade was adopted to scrape tumor cells from the entire slide.^18^

For cytology slides containing tumor cells with sparse small colonies, we used the cell transfer technique and conducted microdissection with slight modification.^19^, ^20^ Briefly, (a) removed slide glass aforementioned method, (b) the cytological materials were covered with a layer of a mixture of xylene and marinol (1:1) medium; (c) the medium was solidify by placing the slides in a oven at 70°C for 30 minutes; (d) put the slides at room temperature for 1 hour; (d) the slides were soaked in 40°C warm water for 10 minutes; (e) using scalpel blade, the medium membrane with its attached cells was peeled off from the slides. The lifted membrane was put on Arcturus PEN membrane glass slides for laser‐capture microdissection system (Thermo Fisher Scientific Waltham, MA).^21^

### 2.3 DNA extraction, and mutation analysis by Next‐Generation Sequencing (NGS)

DNA was extracted using a GeneRead DNA FFPE Kit (Qiagen, Hilden) as described previously.^21^, ^22^, ^23^ DNA quality was checked using two sets of primers targeting the ribonuclease P (RNase P) locus.^21^, ^22^, ^23^, ^24^, ^25^, ^26^ The matched peripheral blood samples were collected from each patient. Buffy coat was isolated following centrifugation, and DNA was extracted from the buffy coat using a QIAamp DNA Blood Mini Kit with a QIAcube system (Qiagen). Construction NGS library for targeted sequencing was conducted as described previously.^25^ The library concentration was determined using an Ion Library Quantitation Kit. Emulsion PCR and chip loading were carried out on the Ion Chef with the Ion PI Hi‐Q Chef kit. Sequencing was conducted on the Ion Proton Sequencer (Thermo Fisher Scientific). Sequence data analysis was carried out as described previously.^21^, ^22^, ^23^, ^25^, ^26^ Actionable mutations were referred to the OncoKB database (update: June 21, 2019) from the Memorial Sloan Kettering Cancer Center.^27^ The VAF represents proportion of tumor cells harboring the specific mutation assuming a good quality tumor samples with high tumor purity.^28^, ^29^ Therefore, in this study we defined “clonal mutation” as mutations with a higher clonal burden (VAF ≧ 10%) and annotated as oncogenic or likely oncogenic by OncoKB database.^28^ All other mutations than clonal mutations are defined as “subclonal mutation”.

### 2.4 Oncomine DX target test

Oncomine Dx Target Test Multi CDx System was approved by Ministry of Health, Labor and Welfare for companion diagnostic test and covered by the health insurance in Japan at June at 2019. We conducted the Oncomine Dx Target Test for research use using cytological specimens from three lung cancer patients who harbored *ALK* and *ROS1* fusion genes detected by orthologous companion diagnostic kits. The panel included 23 genes: *AKT1*, *ALK*, *BRAF*, *CDK4*, *DDR2*, *EGFR*, *ERBB2*, *ERBB3*, *FGFR2*, *FGFR3*, *HRAS*, *KIT*, *KRAS*, *MAP2K1*, *MAP2K2*, *MET*, *MTOR*, *NRAS*, *PDGFRA*, *PIK3CA*, *RAF1*, *RET*, and *ROS1*.^30^ We performed Oncomine Dx Target Test, sequencing on Ion Torrent PGM Dx platform and analyzed data on Torrent Suite Dx Software according to the manufacture's protocol.

### 2.5 Immunohistochemistry (IHC) of ALK proteins

To examine the ALK protein expression, 3 µmol/L‐thick FFPE tumor tissue was stained by IHC. We used an anti‐ALK antibody in Histofine ALK iAEP kit (Nichirei Bioscience, Tokyo, Japan) manually or Ventana OptiView ALK (D5F3) kit (Roche, Tucson, AZ) combination with the OptiView DAB IHC detection and OptiView Amplification (AMP) kits using the Ventana BenchMark XT staining system (Roche).^31^ The AL‐positive staining was evaluated by pathologists (TO) when strong cytoplasmic granular staining was observed only in tumor cells.

### 2.6 *ROS1* fusion gene

The sliced FFPE tumor sections were analyzed by SRL Inc using an in vitro diagnostic AmoyDx *ROS1* Fusion Gene Detection Kit (Amoy Diagnostics Co., Ltd) according to manufacturer's protocol. In brief, total RNA was extracted from FFPE tissue using RNeasy FFPE kit according to manufacturer protocol (Qiagen). Reaction samples were mixed with 18.5 μL of *ROS1* Reverse Transcription Mixture, 0.5 μL of *ROS1* Reverse Transcription Enzyme and 6 μL of total RNA. PCR condition is as follows: 42°C, 1 hour and 95°C, 5 minutes. Complementary DNA (cDNA) were subjected to multiplex RT–PCR as follows: 95°C, 5 minutes, 15 cycles of 95°C, 25 seconds, 64°C, 20 seconds and 72°C, 20 seconds followed by 31 cycles of 93°C, 25 seconds, 60°C, 35 seconds, and 72°C, 20 seconds.

### 2.7 Statistical analysis

The R commander named EZR was used for statistical analysis such as two‐sample t‐test, Kruskal‐Wallis rank sum test, and Pearson's chi‐squared test.^32^

## 3 RESULTS

### 3.1 Mutations with higher variant allele frequency were shared tumor and cytology samples

**Figure 1:**
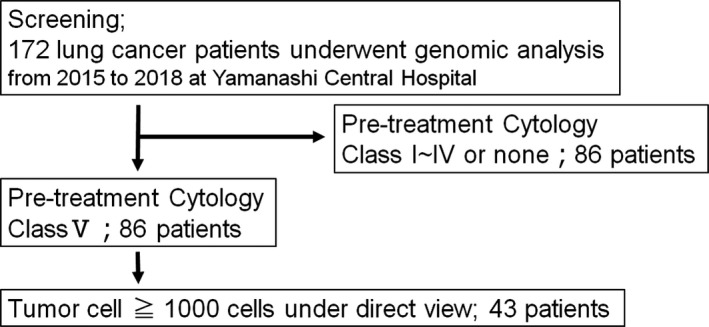
Flowchart of patient selection

Of the consecutive 172 lung cancer patients, whose genomic mutation status of relevant 53 genes associated with lung cancer were analyzed with tumor tissue samples, 86 patients had pretreatment class V cytology samples. Of these 86 patients, we analyzed 46 patients, whose cytology samples had sufficient tumor cells (more than 1,000 tumor cells) (Figure 1). The characteristics of them in two groups are shown in Table 1. The DNA of a total of 43 cytology samples were extracted and analyzed by targeted sequencing of 53 genes. Patient‐matched buffy coats were used as normal controls to detect somatic mutations in each sample.

**Table 1:** Patient characteristics

	All patients (n = 172)	Analysis patients (n = 43)
Age, median	71 [44‐90]	71 [44‐84]
Sex		
Male	116 (67.4)	30 (69.8)
Female	56 (32.6)	13 (30.2)
Stage		
I	79 (45.3)	16 (37.2)
II	21 (12.2)	2 (4.7)
III	33 (19.2)	8 (18.6)
IV	39 (23.3)	17 (39.5)
Histology		
Ad	103 (59.9)	21 (48.8)
Sq	40 (23.3)	10 (23.3)
Ad‐Sq	4 (2.3)	2 (4.7)
NSCLC (NOS)	15 (8.7)	7 (16.3)
SCLC	8 (4.6)	1 (2.2)
SCLC‐Sq	2 (1.2)	2 (4.7)
Cytology		
None	22 (12.8)	0
Brush	105 (61.0)	34 (79.1)
FNA	10 (5.8)	4 (9.3)
Bronchial wash	16 (9.3)	2 (4.6)
Pre‐operative PE	19 (11.1)	3 (7.0)
Class		
I	38 (25.3)	0
II	12 (8.0)	0
III	8 (5.3)	0
IV	6 (4.1)	0
Ⅴ	86 (57.3)	43 (100)

**Figure 2:**
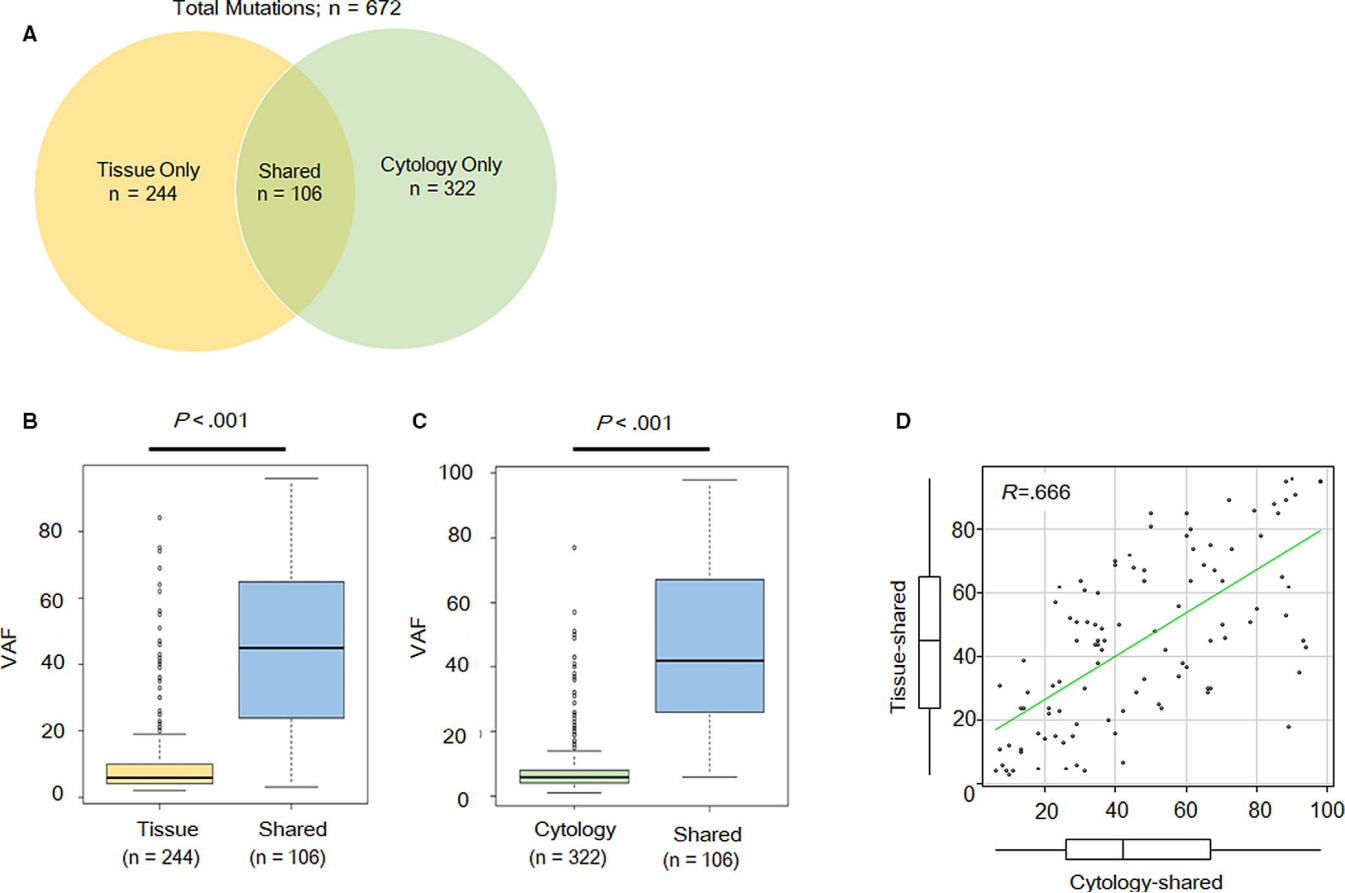
A, Fraction of shared versus tissue or cytology only mutations. B, Variant allele fractions (VAF) of identified mutations in tissue samles. C, VAF of identified mutations in cytology samples. D, A scatterplot graph and partial correlation (*R* = 0.666) analysis results for VAF of shared mutations in cytology samples (*X* axis) and in tissue samples (*Y* axis)

A total of 672 mutations were detected from the peeling samples of archival cytology and corresponding tumor tissue samples. Of the 672 mutations, 106 mutations (15.8%) were shared with both samples, and 244 mutations (36.3%) were detected only in tumor tissue sample and 322 mutations (47.9%) were detected only in archival cytology sample (Figure 2A). The VAF of shared mutations were significantly higher than those mutations which were detected only in either sample (Figure 2B,C). Mean VAF of shared mutations in tumor tissue and in cytology were 45.0% [range: 3%‐96%] and 47.2% [range: 6%‐98%], respectively. Mean VAF of mutation detected only in tumor tissue or in cytology were 11.1% [2%‐84%] and 8.4 [1%‐87%], respectively. The VAF of shared mutations had a moderate positive correlation between tumor tissue and archival cytology samples (Figure 2D).

### 3.2 Genome analysis of archival cytology is useful for detecting clonal mutation in tumor

**Figure 3:**
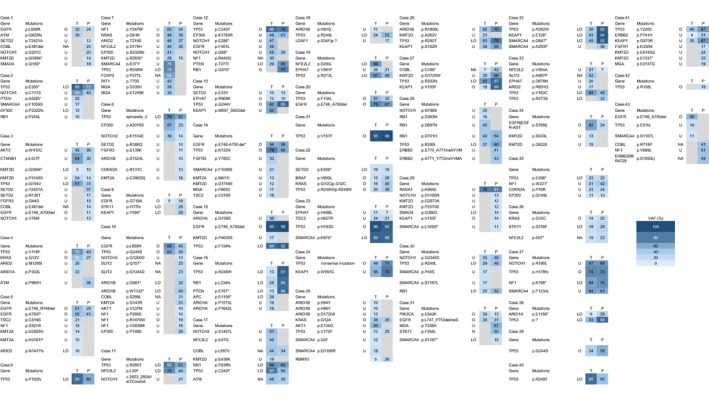
Mutation profiles identified in tumor samples (T) and peeling samples of archival cytology (P). Heat maps show identical mutations in the indicated samples corresponding with primary tumor mutations. Variant allele fraction values are shown in blue with white letters (high value) and light blue with black letters (low value) boxes. Gray boxes indicate no identified mutation in each sample

The comparison heatmap of detected mutations with VAF more than 10% from peeling samples of cytology and those from the corresponding tumor tissues, is shown in Figure 3. Notably, 41 of 43 (95.3%) patients shared at least one mutation. In two cases (case 42 and 43), the genomic data of tumor tissue sample and peeling archival cytology were completely different from each other. Of total 202 mutations whose VAF were more than 10%, the rate of oncogenic (O) or likely oncogenic (LO) mutations was significantly higher in shared mutations than other mutations (60/77 [77.9%] mutations) (Table 2). Majority of mutations detected only in tumor tissue or cytology was nononcogenic or preannotated (84/101 [83.2%] mutations). Therefore, shared and higher VAF (≧10%) mutations have a tendency to be oncogenic. Clonal mutation was defined as mutations with a higher clonal burden (VAF ≧ 10%) and oncogenic or likely oncogenic annotated by OncoKB database.^27^ All other mutations than clonal mutations are defined as subclonal. The majority of clonal mutations in tumor tissue samples were also present in cytology samples. By contrast, subclonal mutations were less likely to be shared in cytology samples. These results showed genome profiling of archival cytology samples identified functionally relevant clonal mutation in lung cancers.

**Table 2:** The number of clonal mutations

Mutations VAF ≧ 10%	Shared	Tissue only	Cytology only	Total	
O/LO mutations (%)	60 (77.9%)	6 (7.8%)	11 (14.3%)	77 (100%)	*P *< .001
Other mutations (%)	41 (32.8%)	53 (42.4%)	31 (24.8%)	125 (100%)	
Total	101	59	42	202	

### 3.3 Actionable driver mutations in archival cytology samples

To examine the clinical utility of genomic analysis of peeling archival cytology samples, we referred to the OncoKB database^27^ and searched actionable driver mutations. A total of 19 actionable driver mutations were detected in 19/43 (44.2%) patients (Table 3). Three actionable driver mutations were only in either cytology or tissue samples. *EGFR* exon 19 deletion (ex 19 del) mutation in Case.43 was detected only in tumor sample and *EGFR* ex 19 del in Case.3 and *NF1* R1870W in Case.10 mutations were detected only in peeling archival samples, respectively. All other 16 mutations (84.2%) were detected from both samples (Table 3). *EGFR* mutations in tyrosine kinase coding region are actionable mutations, for which molecular targeted drugs; EGFR‐tyrosine kinase inhibitors are now available. Genomic analysis of peeling archival cytology could successfully detect 9/10 (90%) *EGFR* mutations, except for Case.3 in which *EGFR* mutation did not be detected from tumor tissue sample. Peeling sample of archival cytology can yield driver mutation profile of the patient.

**Table 3:** List of actionable driver mutations

Case ID	Gene	Mutations	Tumor	Peeling cytology	Cancer type	Drugs
Case.1	*EGFR*	L858R	+	+	NSCLC	Erlotinib
Case.10		L858R	+	+		Afatinib
Case.3		Ex.19 deletion	−	+		Osimertinib
Case.5		Ex.19 deletion	+	+		Dacomitinib
Case.14		Ex.19 deletion	+	+		Gefitinib
Case.20		Ex.19 deletion	+	+		
Case.31		Ex.19 deletion	+	+		
Case.43		Ex.19 deletion	+	−		
Case.5	*EGFR*	A750P	+	+	NSCLC	Erlotinib
Case.9		G719A	+	+		Afatinib
Case.34		S768I	+	+		Gefitinib
Case.4	*KRAS*	G12V	+	+	All Solid Tumors	Cobimetinib
						Binimetinib
						Trametinib
					Colorectal Cancer	Panitumumab
						Cetuximab
Case.25	*KRAS*	G12A	+	+	Colorectal Cancer	Panitumumab
Case.22	*KRAS*	G12C	+	+		Cetuximab
					Histiocytosis	Cobimetinib
					All Solid Tumors	Cobimetinib
						Binimetinib
						Trametinib
Case.7	*NRAS*	Q61K	+	+	Colorectal Cancer	Panitumumab
						Cetuximab
					Tyroid Cancer	Iodine + Selumetinib
					Melanoma	Binimetinib
						Binimetinib + Ribociclib
					Histiocytosis	Cobimetinib
Case.10	*NF1*	R1870W	−	+	All Solid Tumors	Cobimetinib
						Trametinib
Case.12	*PTEN*	T277I	+	+	All Solid Tumors	GSK 2636771
Case.16	*PTEN*	E167*	+	+		AZD8186
Case.31	*PIK3CA*	E542K	+	+	Breast Cancer	Alpelsib + Fulvestrant

* Truncating mutation.

### 3.4 *ALK* and *ROS1* fusion genes were detected in cytological sample

We searched the *ALK* or *ROS1* fusion‐positive samples retrospectively. Three samples were previously analyzed by orthologous kit and determined to be *ALK* fusion‐positive (n = 2) by IHC and *ROS1* fusion‐positive (n = 1) by RT‐PCR using FFPE tumor tissues (Table S1, Figure S1). Along with the FFPE tumor tissues, patient‐matched archival cytological samples were also available for genomic analysis. To examine the fusion gene could be detected from cytological sample, we extracted total RNA from three cytological samples and analyzed by companion diagnostic kit approved in Japan, Oncomine Dx Target Test. As a result, the same fusion genes could be detected in two *ALK* fusion gene‐positive samples and one *ROS1* fusion gene‐positive sample, respectively (Table S1). These results suggested actionable fusion genes could be detected by genomic analysis of archival cytological samples from lung cancer patients.

### 3.5 Sequential analysis of cytology samples could detect the change in driver mutations

**Figure 4:**
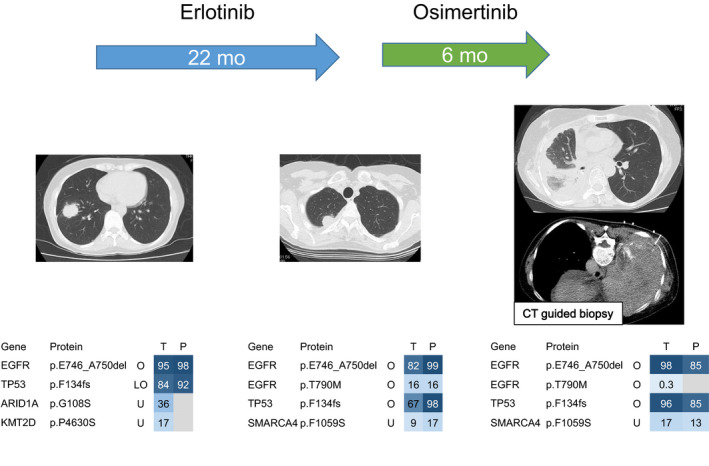
Clinical course and mutation profiles of identified in primary tumor (T) and correspondent peeling of cytology samples (P). Chest CT scan images show the primary tumor and heat maps show identical mutations in the indicated samples, which were obtained at the CT scan images, respectively. Variant allele fraction values are shown in blue (high value) and light blue (low value) boxes. Gray boxes indicate no identified mutation in each sample

Sequential archival cytology samples also well reflect the genomic information of the corresponding tumor tissue samples (Figure 4). A 52‐year‐old Japanese woman (Case.15 in Figure 3) was diagnosed as advanced lung adenocarcinoma harboring *EGFR* ex 19 del and *TP53* p.F134fs mutations by the analysis of the sample of the first bronchial biopsy. Erlotinib was administered as the first‐line chemotherapy. After 22 months of erlotinib treatment, the tumor recurred and the second bronchial biopsy was performed. The genomic analysis of the tumor tissue and the correspondent peeling of archival cytology sample revealed the same clonal mutation profile. The second genomic analysis revealed *EGFR* T790M mutation and osimertinib was administered as the second‐line chemotherapy (Figure 4). After 6 months of osimertinib treatment, the tumor relapsed and the third biopsy was performed by the CT‐guided biopsy. The third genomic analysis of the tumor tissue and the correspondent peeling of fine‐needle aspiration cytology sample successfully revealed the same clonal mutation profile (Figure 4). The VAF of *EGFR* T790M mutation was reduced after osimertinib treatment and might reflect the antitumor effect of this drug. In all three genomic analyses of tumor tissues and correspondent peeling samples, the results of genomic profile were similar. These results indicated serial genome analysis of cytological specimen revealed the dynamic changes in resistant mutation during the course of EGFR‐TKI treatment.

## 4 DISCUSSION

The rapid growth of advance in molecular profiling of lung cancer has been developing personalized medicine and precision medicine especially in the treatment of advanced lung cancer patients. How we successfully obtain genomic information from limited quantities of tumor tissue samples becomes a central issue. Furthermore, repetitive sampling of tumor is necessary for analyzing the changing genomic profile in reaction to chemotherapy especially including molecular targeted therapies.^33^, ^34^ However, in advanced lung cancer patients, repetitive tumor biopsy sufficient for NGS analysis is often challenging because surgical biopsy is too invasive for frail patients with advanced cancer.^9^ Cytology samples have the potential to be an alternative to tumor tissue samples for NGS analysis.^11^, ^12^ In this study, we show that the tumor–cell‐rich archival cytology samples can yield genomic information of primary lung tumor including driver mutation profile.

Genomic analysis of peeling archival cytology samples can yield the information of actionable driver mutations. Genomic profiling of lung cancer by The Cancer Genome Atlas (TCGA) has demonstrated that up to 90% of cancer patients harbor at least one potentially actionable driver mutation.^35^ Multicenter genomic testing has revealed that up to 64% of NSCLC patients have an actionable driver mutation.^36^ For those NSCLC patients who have a actionable driver mutation, improved overall survival was observed among those treated with vs not treated with targeted therapies (median, 18.6 months vs 11.4 months from advanced diagnosis; *P * <  .001).^37^ To determine the best treatment approach based on genomic profile before treatment leads to improved prognosis of the advanced NSCLC patients. In our patient cohort, actionable diver mutations which have corresponding available drugs were *EGFR* major mutations (ex 19 del, G719A, A750P, S768I, and L858R) and *ALK*, *ROS1* fusion genes. In Case.3, *EGFR* ex 19 del mutation was detected only in tumor sample and peeling cytology sample did not yield the mutation. Except for the case, NGS analysis of peeling cytology samples could detect *EGFR* major mutations at a frequency as high as 90%. RNA was also successfully extracted from peeling cytology samples and well feasible for NGS analysis. AMG 510 is the first small molecule inhibitor to successfully target KRAS mutation in advanced cancer patients, according to findings presented at the 2019 American Society of Clinical Oncology Annual Meeting.^38^ *KRAS* mutation is the major driver mutation in NSCLC patients second to *EGFR* mutation. In our cohort, Case.22 had *KRAS* G12C mutation detected from the tumor tissue sample, which was replicated by the analysis of peeling cytology sample. There is accumulating evidences of next coming actionable driver mutations including MET exon14 mutation, RET fusion gene, NTRK fusion gene, FGFR gene amplification, and so on.^3^, ^39^ When tumor tissue samples were exhausted for investigating existing actionable driver mutations, archival cytology samples can compensate for the lost.

We showed a successful case, in which the genomic profile of sequential archival cytology samples well reflected that of the correspondent tumor tissues (Figure 4). It is often difficult to obtain serial tumor tissue samples in patients with advanced lung cancer.^9^ Biopsy is often not possible, particularly in central lesions, and cerebrospinal fluid cytology is often the only diagnosis. However, if there are sufficient tumor cells by cytology, as in the present case, it may well reflect the genome information of the correspondent tumor itself, and it may be possible to analyze the genetic evolution of cancer in the course of treatment using sequential cytology samples including a cerebrospinal fluid cytologic sample.

The complete discrepancy in the result between tumor tissue and cytology samples was found in 2/43 (4.7%) patients (case 42 and 43). In three cases (Case.3, 10, 43), driver mutations were detected in only one sample. In these cases, we should administer the corresponding targeted therapies. A large clinical database revealed that overall survival was improved among NSCLC patients those who were treated with targeted molecular therapies.^37^ In major cases, driver mutations were shared between tumor tissue and cytology samples. It is not necessary to explore driver mutations by analyzing both samples for costing time and money. In Case 3, *EGFR* ex 19 del mutation, an important targetable driver mutation, was detected only in the cytology sample. The relatively low VAF of the mutation in the cytology sample (11%) may be due to the intratumoral heterogeneity of the mutation,^40^ because the tumor tissue was microdissected and sequenced. Alternatively, the formalin‐fixation of the tumor tissue sample resulted in degradation of DNA and decreased sensitivity for the detection of EGFR ex.19 deletion mutation. Generally, tumor tissue samples, especially surgical samples contain more tumor cells with good condition than cytology samples do. In searching for actionable driver mutations, we should give priority to tumor tissue samples over cytology samples. However, from this study, archival cytology samples can yield the genetic information comparable to tumor tissue sample as long as the mutations are limited to clonal mutations.

Peeling sample of archival cytology has some advantages over FFPE tumor samples. 1) The preparation time of peeling samples for NGS analysis is shorter than that of FFPE tumor samples.^15^, ^18^ The pathological analysis of cytology samples takes only one day. On the other hand, the pathological analysis of FFPE tumor samples takes a few days including section for mounting on a slide and hematoxylin and eosin staining. For urgent cases who need the genomic information of their tumors as soon as possible, cytology sample containing plenty of tumor cells can be used for NGS analysis. 2) Diff‐Quick stained cytology samples can yield better quality of DNA. The formalin used for making FFPE tumor samples or the hematoxylin in the Papanicolaou stain are not necessary for Diff‐Quick stained cytology samples, which degrade nucleic acids.^13^, ^41^, ^42^

There are some limitations in this study. First, we selected class V and tumor–cell‐rich cytology samples and did not investigate the utility of class I‐IV cytology samples. The class I and II cytology samples are not promising because there are apparently no tumor cells. However, class III and IV cytology samples have the possibility that they yield genetic information of primary tumor. To clarify the utility of archival cytology samples, the peeling samples of class III and IV cytology slides should be investigated whether they can be the reliable resource of genetic information. Second, this is the retrospective design of study and there is a case selection bias. Third, we used only archival cytology samples in this study. Fresh cytology samples without ethanol‐ or methanol‐fixed procedure from fluid samples obtained from bronchoscopy would yield higher quality DNA and RNA. Fresh cytology samples do not need peeling off procedure and are easier to obtain genetic information than archival cytology samples. However, they have a disadvantage in that we cannot detect how many tumor cells they contain. Forth, we analyzed limited number of samples were analyzed in retrospective study. Although analyzed samples were relatively small, the data indicated the genomic profiles in cytological samples reflected that of FFPE samples in NSCLC. To reinforce the utility of the archival cytology samples, further study is needed in larger scale cases.

## 5 CONCLUSIONS

We show archival cytology sample with adequate tumor cells can yield genetic information of the correspondent primary tumor through NGS analysis. If tumor tissue samples are exhausted, we can use archival cytology samples to search for actionable driver mutations.

## CONFLICT OF INTEREST DISCLOSURES

The authors had no disclosures associated with this study.

## AUTHORS’ CONTRIBUTIONS

Kei Kunimasa and Yosuke Hirotsu involved in conceptualization, data curation, investigation, methodology, and writing‐original draft. Kenji Amemiya and Yuki Nagakubo involved in data curation, investigation, and methodology. Taichiro Goto performed investigation, methodology, and writing‐review and editing. Yoshihiro Miyashita, Yumiko Kakizaki, Toshiharu Tsutsui, Sotaro Otake, Hiroaki Kobayashi, Rumi Higuchi, Kie Inomata, Takashi Kumagai participated in investigation. Hitoshi Mochizuki carried out data curation and methodology. Harumi Nakamura, Toshio Oyama, and Shin‐ichi Nakatsuka involved in methodology. Kazumi Nishino, Fumio Imamura, and Toru Kumagai involved in writing‐review and editing of the manuscript. Masao Omata performed conceptualization, methodology, and writing‐review and editing.

## ETHICAL APPROVAL

Informed consent was obtained from all patients. This study was approved by the Institutional Review Board at Yamanashi Central Hospital.

## Supporting information

Fig S1

Table S1
