# Supplementary figures and images for: Genome analysis of peeling archival cytology samples detects driver mutations in lung cancer
Source: Cancer Med. 2020 Apr 29;9(13):4501–11. doi: 10.1002/cam4.3089 (PMC7333826; doi:10.1002/cam4.3089)

## Slide 1
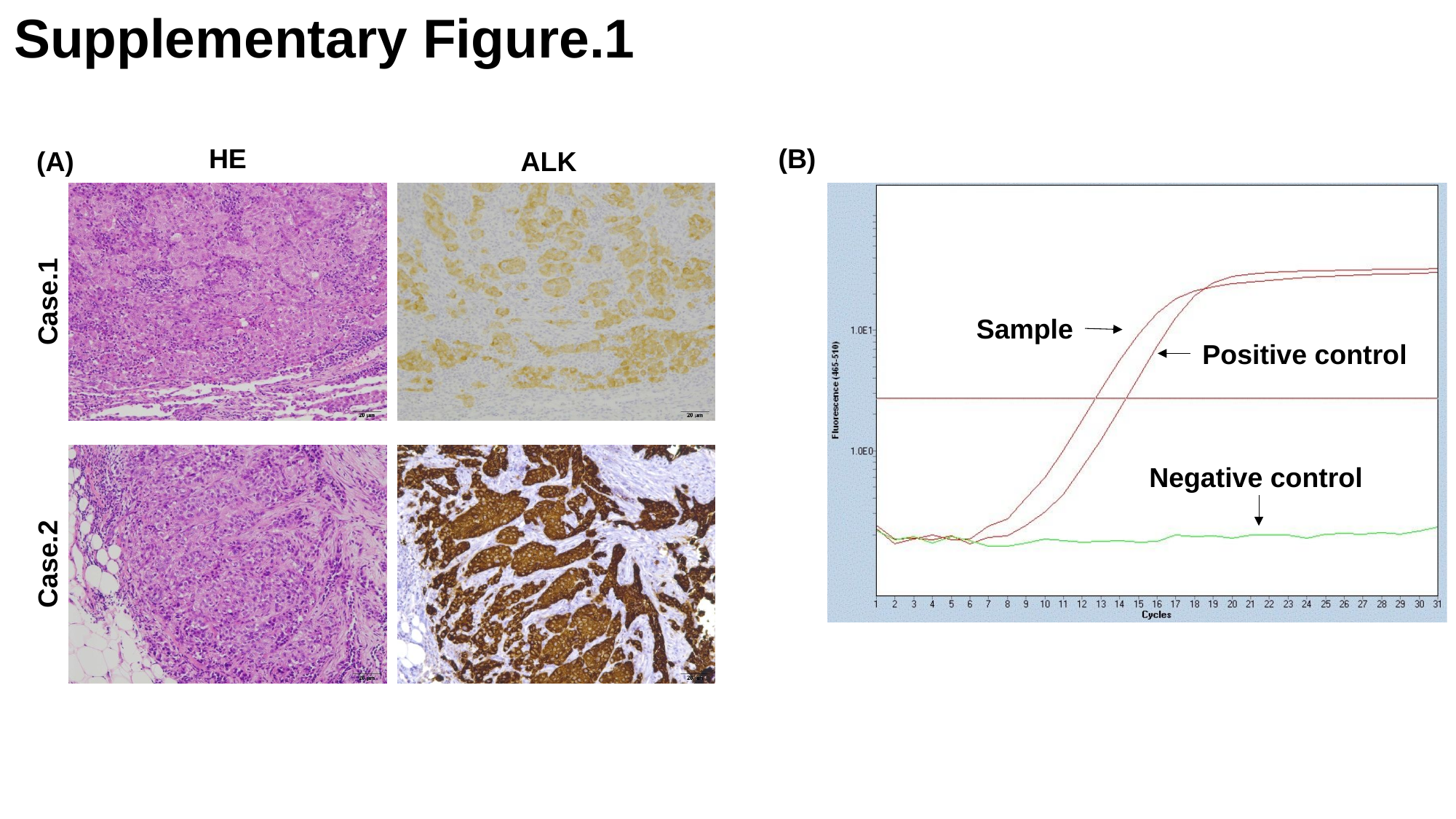

Supplementary Figure.1
HE
(B)
(A)
ALK
Sample
Positive control
Negative control
Case.1
Case.2

Supplement: Supplementary file 1 — Fig S1 [file CAM4-9-4501-s001.pptx]
