# Supplementary material for: Genome analysis of peeling archival cytology samples detects driver mutations in lung cancer
Source: Cancer Med. 2020 Apr 29;9(13):4501–11. doi: 10.1002/cam4.3089 (PMC7333826; doi:10.1002/cam4.3089)
Supplement: Supplementary file 2 — Table S1 [file CAM4-9-4501-s002.docx]

Supplementary Table.1 List of *ALK*, *ROS1* fusion gene positive patients
